# Financial, Legal, and Functional Challenges of Providing Care for People Living With Dementia and Needs for a Digital Platform: Interview Study Among Family Caregivers

**DOI:** 10.2196/47577

**Published:** 2023-09-05

**Authors:** Qiping Fan, Logan DuBose, Marcia G Ory, Shinduk Lee, Minh-Nguyet Hoang, Jeswin Vennatt, Chung Lin Kew, David Doyle, Tokunbo Falohun

**Affiliations:** 1 Department of Health Behavior School of Public Health Texas A&M University College Station, TX United States; 2 Department of Public Health Sciences Clemson University Clemson, SC United States; 3 Internal Medicine George Washington University Washington, DC United States; 4 Department of Environmental and Occupational Health School of Public Health Texas A&M University College Station, TX United States; 5 Division of Health Systems and Community-Based Care College of Nursing University of Utah Salt Lake City, UT United States; 6 School of Medicine Texas A&M University College Station, TX United States; 7 College of Medicine Central Michigan University Mt Pleasant, MI United States; 8 Department of Biomedical Engineering Texas A&M University College Station, TX United States

**Keywords:** family caregiver, Alzheimer disease, dementia, caregiving challenges, digital health, community-based participatory research, mobile phone

## Abstract

**Background:**

Alzheimer disease and Alzheimer disease–related dementia represent complex neuropathologies directly challenging individuals, their families, and communities in the United States. To support persons living with dementia, family or informal caregivers often encounter complex financial, psychological, and physical challenges. A widely used solution such as a consolidated web-based assistance or guidance platform is missing, compounding care challenges.

**Objective:**

In preparation for designing an internet-based artificial intelligence–driven digital resource platform, a qualitative interview study was conducted to characterize the challenges and needs of family caregivers in the United States.

**Methods:**

A semistructured interview topic guide in English was developed by engaging community partners and research partnerships. Family caregiver participants were purposefully recruited via various means, such as word of mouth, local dementia community service providers, digital recruitment emails, flyers, and social media. Interested individuals were first invited to complete an eligibility screening survey, and eligible individuals were then contacted to arrange a web-based in-depth interview via Zoom (Zoom Video Communications) from January 1, 2022, to May 31, 2022. A follow-up survey was administered in May 2022 to provide an overview of the participants’ demographics, socioeconomic characteristics, and caregiving information. Thematic analysis in a framework approach was used to identify and organize themes and the study findings.

**Results:**

Following the prescreening of 150 eligible respondents, 20% (30/150) individuals completed both the interviews and follow-up survey, allowing for an in-depth look into the challenges, experiences, and expectations of primary caregivers of people living with dementia. Most participants (20/30, 67%) were primary caregivers of persons with dementia, and 93% (28/30) had provided care for at least a year. Most participants were aged >50 years (25/30, 83%), female (23/30, 77%), White (25/30, 83%), and non-Hispanic (27/30, 90%) and held a bachelor’s or graduate degree (22/30, 73%). Collectively, all participants acknowledged challenges in caring for people living with dementia. Thematic analyses elicited the challenges of caregiving related to functional care needs and financial and legal challenges. In addition, participants identified the need for an integrative digital platform where information could be supplied to foster education, share resources, and provide community support, enabling family caregivers to improve the quality of care and reducing caregiver burden.

**Conclusions:**

This study emphasized the difficulties associated with the family caregiver role and the expectations and potential for a supportive web-based platform to mitigate current challenges within the caregiving role.

## Introduction

### Background

Approximately 6.5 million Americans aged ≥65 years live with Alzheimer disease (AD) and AD-related dementia (ADRD)—a debilitating and progressive neurocognitive disease leading to the loss of memory and motor function and other negative psychosocial symptoms [1,2]. The number of persons living with AD or ADRD is expected to grow to 13.8 million by 2060, calling for innovative solutions to address this increasing public health crisis [1]. Persons living with dementia often require many aspects of care, including dressing and support with daily activities as well as arranging for specialized health professionals such as nurses for personal care, advisors for legal issues, or accountants for financial assistance [3]. Most people living with dementia are cared for by family or informal caregivers, and the largest proportion of these caregivers comprises spouses, followed by children and children-in-law, with most being female [3]. The caregivers of people living with dementia face a significant burden considering that these caregivers themselves are often older or juggling their own professional and other family responsibilities [3]. To combat the increasing public health burden, the National Plan to Address Alzheimer’s Disease called for enhancing support for people living with dementia and their families as well as further funding for AD research and the development of effective interventions [4,5].

The family caregivers of people living with dementia provide the vast majority of direct care and care management, and they face a high degree of psychological, physical, and financial stress [1]. These caregivers are most often family members or friends (eg, unpaid, family, or informal caregivers) [6]. There are >11 million family caregivers of people living with dementia in the United States providing an aggregate of >16 billion hours annually, with an estimated economic cost of US $271.6 billion for their care needs in 2021 alone [1]. These family caregivers are at greater risk of developing depression, anxiety, social isolation, and physical health problems because of the chronic stress associated with caregiving [6,7]. Moreover, caregivers encounter various additional challenges in their role, including difficulties in accessing relevant caregiving services and information, dissatisfaction with the quality or lack of trust in available services, and the inflexibility of existing service options [8]. Although previous research has highlighted general caregiving challenges, there is a lack of studies that specifically explore the financial (eg, where to find assistance if there are difficulties in paying for caregiving services), legal (eg, issues of guardianship), and functional (eg, assistance in meeting activities of daily living) difficulties experienced by family caregivers of people living with dementia. Therefore, it is imperative to highlight the importance of enhancing family caregiving support and gaining a comprehensive understanding of caregivers’ challenges. Gaining a comprehensive understanding of caregivers’ challenges and needed supports is essential for deriving real-world, practical solutions to improve caregivers’ quality of life and, in turn, that of their care recipients living with dementia [9].

Several studies have suggested the importance of innovative technological solutions for empowering and supporting people living with dementia and their family caregivers [10,11]. Digital tools have presented promising means to assist family caregivers in seeking information to improve the care they provide to people living with dementia [12]. However, the information provided by currently available digital search engines and web-based educational resources is not always easily accessible or useful and is rarely individualized or tailored to the diverse needs of family caregivers. There is a need for more theory-driven digital tools to assist caregivers [13] and involve them in developing and designing digital health solutions [14]. A conceptual model derived from a web-based caregiver forum suggested that understanding individuals with ADRD, their caregivers, the caregiver–individual with ADRD dynamic, and the context of care is essential to improve care for individuals with AD or ADRD and their caregivers [15]. Future programs, tools, and services developed must involve caregivers early and be both accessible and tailored to caregivers’ specific needs, which can, in turn, reduce the caregiving burden [7,16].

### Objectives

Considering the need for caregiver-centered assistance tools, and in preparation for designing an interactive web-based artificial intelligence (AI)–driven digital resource platform, this study aimed to investigate (1) the legal, financial, and functional challenges that caregivers face in providing care for people living with dementia and (2) their expectations for the features of a digital health platform that assists in identifying and accessing the legal, financial, and functional care support and services needed to provide quality care and reduce caregiver burden.

## Methods

### Overview

**Figure 1:**
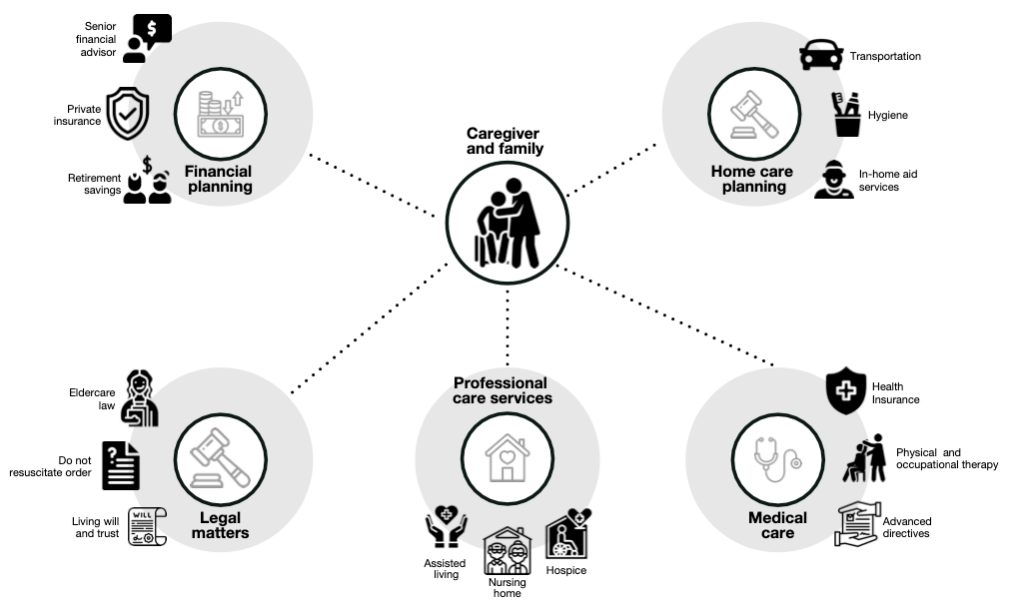
Aspects of caregiving for individuals with Alzheimer disease or Alzheimer disease–related dementia.

In this study, a community-engaged research framework [17] was used to engage both researchers and communities of people living with dementia to identify and define health problems, determine the research questions asked, interpret results, develop interventions to address public health problems, and disseminate the results of the study. By engaging community members and researchers in understanding the key aspects of caregiving for individuals with AD and ADRD (Figure 1), a semistructured interview topic guide was developed.

The interview topic guide was formulated by leading researchers after considering the priorities expressed by relevant stakeholders who were part of our preliminary formative work. Pretesting of the interview topic guide was conducted with multiple caregivers. The final version (Multimedia Appendix 1) was achieved through collaborative discussions involving caregivers, community partners, and researchers. The following are some illustrative interview questions aimed at exploring the challenges and experiences related to caregiving as well as the expectations for a web-based tool designed to assist caregivers: (1) What was the most challenging aspect of finding and securing living arrangements or financial services or legal services for your care recipient? (2) Did you consult with any professionals to help you with caregiving responsibilities? If not, why not? If so, what kind of professionals and how did you select them? and (3) If you could have a “magic wand” to create a web-based tool or platform that would assist you with your caregiving, what kind of features would it have?

The findings of this study will contribute to the design of a digital health platform funded by the National Institute on Aging through the Small Business Innovation Research program aimed at enhancing caregiving support.

### Study Setting

**Figure 2:**
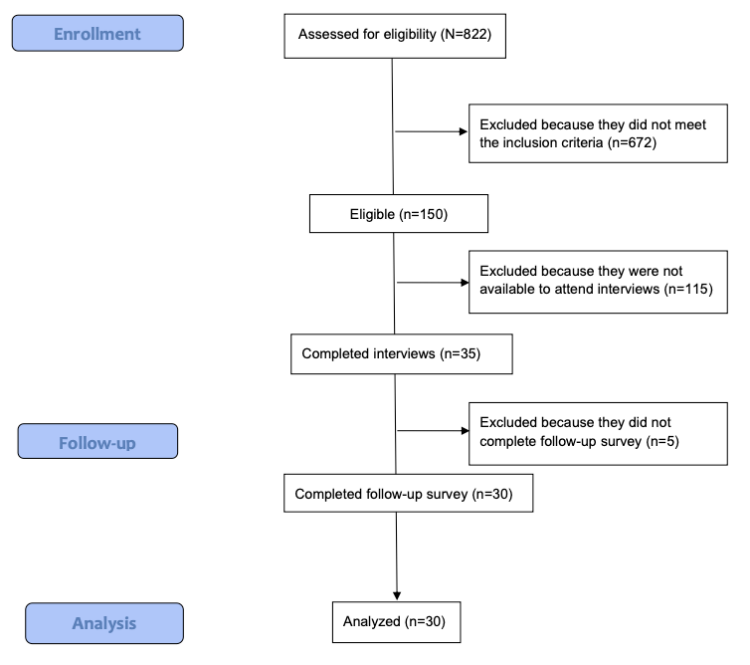
Study flow diagram.

This qualitative study was conducted between January 1, 2022, and May 31, 2022. This study adopted a purposeful sampling method to recruit caregivers of people living with dementia through local dementia community service providers; word of mouth; and web-based approaches such as recruitment emails, flyers, and social media (eg, Facebook and LinkedIn). The sample size of the study was determined based on data saturation, referring to the stage at which further data collection is unlikely to provide novel information or alter the existing analysis and interpretations in qualitative studies [18]. All in-depth interviews were conducted in English using the Zoom (Zoom Video Communications) web-based platform. The research team consisted of members from diverse cultural, ethnic, and gender backgrounds, bringing together expertise in biomedical engineering, business, medicine, computer science, and public health. The process of enrollment, follow-up, and analysis is presented in the study flow diagram (Figure 2).

### Participants

Individuals who conveyed an interest in the study were invited to complete a web-based screening questionnaire to assess their eligibility and willingness to participate. The questionnaire included an electronic informed consent agreement in English, which provided essential details about the study, including a description of the study objectives and activities, contact information of the study leaders and the institutional review board (IRB) agency that approved the study, confidentiality and privacy, compensation, and instructions on how to indicate voluntary participation.

The inclusion criteria for participants were as follows: individuals aged ≥18 years who were nonpaid caregivers of people living with dementia. These potential participants included adult children, spouses, partners, other family members, or legal guardians of people living with dementia who were actively involved in making legal and financial decisions. Moreover, participants had to be seeking eldercare services within the United States and had to have expressed concerns or a perceived need for more information on financial management and legal planning related to caregiving. In addition, access to a smartphone or computer with internet connectivity was necessary. Conversely, the exclusion criteria ensured that care recipients were living in the community, excluding nursing homes, locked memory care units, or other institutional settings. Furthermore, this study intentionally excluded formal caregivers, such as paid or professional caregivers, to focus solely on the experiences and challenges faced by nonpaid caregivers.

### Procedures

Interested individuals were first invited to complete a web-based eligibility screening survey to assess their eligibility and willingness to participate. Eligible individuals were contacted via email and phone to arrange web-based in-depth interviews conducted in English. The interviews were conducted by study personnel via Zoom video calls that took place between January 2022 and May 2022. The study personnel who conducted the interviews were graduate students who had received training on human participant research and digital health information to familiarize themselves with the study context and participants. The study personnel began each interview with an information session and obtained verbal informed consent from the participants to take part and be recorded. Each interview was guided by the pretested semistructured interview topic guide, lasted approximately 40 to 60 minutes, and concluded by asking participants if they had additional questions. After the participants exited the session, there was often a short debriefing session among the study personnel. The recorded audio of the Zoom meeting in English was first transcribed using either Zoom or Otter.AI, an AI-powered automatic and real-time transcription tool. The transcripts were then cleaned by the study personnel and uploaded to a shared encrypted Dropbox drive (Dropbox, Inc). In May 2022, participants who had completed the interviews were contacted to complete a follow-up survey, which aimed to collect information regarding their demographic characteristics.

### Analysis

Using the qualitative research framework approach [19], thematic analysis was conducted to identify and organize themes and the study findings. The framework approach was developed in the 1980s [20] and is widely used in analyzing qualitative data in health research [19]. This approach involves several key steps, including transcribing interviews, becoming familiar with the interview material, coding, developing an analytical framework, applying the analytical framework, charting data into the framework matrix, and interpreting the data [19]. The researchers read the transcripts to familiarize themselves with the data and developed the codebook in 3 steps. First, the researchers (QF, LD, MNH, and JV) developed a preliminary codebook using the interview topic guide and several randomly selected transcripts. Randomization was conducted by selecting transcripts represented by study ID numbers using analytical software. The researchers then determined a transcript with abundant findings to code together and revised the codebook in a group meeting. Finally, 2 independent coders (QF and MNH) coded 5 transcripts to refine and finalize the codebook with the guidance of experienced eldercare and qualitative research advisors (MGO, SL, and CLK). No new themes or codes came up while coding the remaining transcripts. The 2 coders (QF and MNH) independently coded all the transcripts using the Windows version of the NVivo (version 12.0; QSR International) software. The intercoder reliability was evaluated, with an average agreement rate of 98.52%, indicating almost perfect agreement. The study personnel (QF, LD, MNH, and JV) discussed any disagreements and reached a consensus after a thorough review. After completing the coding process, the researchers (QF and MNH) developed a framework and charted the findings into a framework matrix in a Microsoft Excel sheet (Microsoft Corp). This involved documenting the identified major themes, with subthemes nested under each major theme. For each subtheme, explanations and key quotes from participants reflecting the subtheme were provided.

### Ethics Approval

The research team obtained ethics approval from the IRB at Texas A&M University (IRB approval IRB2021-0943D).

### Informed Consent

The study personnel asked all participants for electronic informed consent in the screening survey and obtained verbal informed consent from participants before they were interviewed.

## Results

### Description of the Sample

Prescreening surveys were completed by 822 respondents, of whom 150 (18.2%) were eligible. Of those 150 eligible respondents, 30 (20%) participated in the in-depth interviews and completed the study survey. The background characteristics of the 30 interview participants are presented in Table 1. More than two-thirds (20/30, 67%) of the participants were primary caregivers for people living with dementia, and 93% (28/30) had provided care for at least 1 year. In total, 40% (12/30) of the participants had provided care for >40 hours a week for the past 3 months.

**Table 1:** Background characteristics of the study participants (n=30).

Background characteristics			Values, n (%)
**Role in providing care for ≥1 adults aged >50 years**			
	Participant is the primary caregiver	20 (67)	
	Someone else is the primary caregiver	4 (14)	
	The participant shared caregiving responsibilities approximately equally with someone else	5 (17)	
	Unknown	1 (3)	
**How long has the participant been providing care or assistance for the care recipients?**			
	At least 6 months but <1 year	2 (7)	
	At least 1 year but <5 years	14 (47)	
	At least 5 years but <10 years	8 (27)	
	≥10 years	6 (20)	
**Over the past 3 months, approximately how many hours per week has the participant provided some form of care for ≥1 adults aged >50 years?**			
	<20	9 (30)	
	20-40	9 (30)	
	>40	12 (40)	
**Participant’s age (years)**			
	35-49	5 (17)	
	50-64	12 (40)	
	≥65	13 (43)	
**Sex**			
	Male	7 (23)	
	Female	23 (77)	
**Race**			
	American Indian or Alaska Native	1 (3)	
	Asian, Native Hawaiian, or Pacific Islander	1 (3)	
	Black or African American	2 (7)	
	White	25 (83)	
	Multiracial	1 (3)	
**Ethnicity**			
	Spanish, Hispanic, or Latino origin or descent	3 (10)	
	Other	27 (90)	
**Highest level of education completed**			
	Some college but no degree	5 (17)	
	Associate degree	3 (10)	
	Bachelor’s degree	8 (27)	
	Graduate degree	14 (47)	
**Employment status**			
	Employed for wages	12 (40)	
	Homemaker or self-employed	4 (13)	
	Unemployed or unable to work	2 (7)	
	Retired	12 (40)	
**General financial status at the end of the month**			
	End up with some money left over	20 (67)	
	Have just enough to make ends meet	7 (23)	
	Does not have enough money to make ends meet	2 (7)	
	Does not know	1 (3)	
**Recruitment channel**			
	Web-based advertisement (eg, Facebook and LinkedIn)	11 (37)	
	Email invitation	13 (43)	
	In-person presentation	2 (7)	
	Personal connection	3 (10)	
	Other	1 (3)	

A total of 83% (25/30) of the participants were aged >50 years. This study had 77% (23/30) female participants and 23% (7/30) male participants. In total, 83% (25/30) of the participants were White; the remainder were American Indian or Alaska Native, Asian, Black or African American, Native Hawaiian or Pacific Islander, or multiracial individuals (5/30, 17%). The participants were 90% (27/30) non-Hispanic and 10% (3/30) Hispanic individuals. Regarding the highest level of education, 47% (14/30) of the participants had a graduate degree, 27% (8/30) had a bachelor’s degree, 10% (3/30) had an associate degree, and 17% (5/30) had attended some college but did not have a degree. Of the 30 participants, 12 (40%) were employed for wages, 4 (13%) were homemakers or self-employed, 2 (7%) were unemployed or unable to work, and 12 (40%) were retired. A total of 67% (20/30) of the participants had some excess money at the end of each month, whereas other participants (9/30, 30%) had just enough to make ends meet or did not have enough to make ends meet. Most participants were recruited through email invitation (13/30, 43%) and web-based advertisements (11/30, 37%), but some were recruited through in-person presentations and personal connections (5/30, 17%).

### Themes and Main Findings

#### Overview

The final codebook (Table 2) consisted of 7 topical codes and 30 secondary codes with specific definitions for each secondary code. On the basis of the codebook and coding results, Table 3 summarizes the findings of the interviews by presenting the major themes, subthemes, and subtheme descriptions.

**Table 2:** Analytic codebook of this study after a 3-step testing procedure

Topical code and secondary code			Definition
**Other caregiving challenges**			
	Caregiving challenges	Any other challenges that caregivers face and do not specifically belong in challenges related to older adult living, financial, and legal services for the care recipient	
**Living arrangements**			
	Had living arrangement challenges	Challenges regarding living arrangements (eg, nursing homes, assisted living, home care, hospice, and postacute care placement) for the care recipient	
	No challenges regarding living arrangements	Statement that living arrangements were simple in their case or description of the ease of living arrangement for the care recipient	
	Services used for living arrangements	Services, professionals, businesses, and facilities that a caregiver used to secure living arrangements for the care recipient	
	Factors for identification and selection	Factors (eg, cost, location, quality, and referral) that influenced identifying and selecting living facilities or at-home care agencies for the care recipient	
	Other	Any other information that was related to older adult living for the care recipient but did not fall into other existing codes	
**Financial**			
	Had financial challenges	Any stated challenges regarding the financial aspects of arranging care for a care recipient (eg, paying for care, accessing finances, and working with financial consultants)	
	No financial challenges	The participant stated that financial considerations for their caregiving experience were simple or not challenging for them. Description of the ease of arrangement of the financial situation of the care recipient.	
	Financial services used	Services, professionals, businesses, and consultants that a caregiver used to navigate any financial responsibilities associated with caring for a care recipient	
	Consulted financial professionals	The participant consulted financial professionals to help them navigate financial aspects of older adult care for the care recipient.	
	Did not consult financial professionals	The participant did not consult financial professionals to help them navigate financial aspects of older adult care for the care recipient.	
	Awareness of benefits and other support	Whether the participant was aware of any additional support or any available social benefits to help pay for care or navigate the financial aspects of caregiving	
	Other	Any other information that was related to the financial aspects of older adult care for the care recipient but did not fall into other existing codes	
**Legal**			
	Had legal challenges	Any stated challenges regarding the legal aspects of arranging care for the care recipient	
	No legal challenges	Description of the ease of arrangement of the legal situation of the care recipient	
	Consulted legal professionals	Confirmation of having consulted legal professionals to help them navigate the legal aspect of older adult care for the care recipient	
	Did not consult legal professionals	Confirmation of having not consulted any legal professionals to help them navigate the legal aspect of older adult care for the care recipient.	
	Advanced directives	The participant’s comments or experiences with setting up advanced directives for the care recipient (advanced directives include 3 categories: living will, power of attorney, and health care proxy)	
	Other	Any other information that was related to the legal aspect of older adult care for the care recipient but did not fall into other existing codes	
**Caregiver support group**			
	Used caregiver support groups	Confirmation that the participant used or participated in any caregiver support groups	
	Did not use caregiver support groups	Confirmation that the participant did not use or participate in any caregiver support groups	
**Educational resources**			
	Educational resources that caregivers used in the past	Any educational topics or resources that participants had used in the past	
**Expected features of a platform**			
	Education	The need for education materials, classes, programs, and peer-reviewed research evidence about caregiving for people living with dementia	
	Caregiver support group	The need for support groups or forums to connect with other caregivers of people living with dementia	
	Communication with health providers about care recipient’s condition	Timely communication with health care providers to ask questions about the care recipient’s condition	
	Information or database to find facilities, providers, or services	Obtaining information to find trustworthy facilities, providers, and services for care recipients	
	Mental health support for caregivers	The need for mental health support features of the digital platform, such as stress or anxiety management, affirmation techniques, or communication with mental health care providers about their own mental health	
	Task-based notifications or reminders	The need for task-based notifications or reminders, such as taking medication, submitting paperwork, and scheduling appointments	
	Quality of the platform	Any other qualities or ideal aspects of a web-based platform that should be included	
	Other	Other features that participants need that should be included in a web-based platform that did not fall into other existing codes	

**Table 3:** Themes, subthemes, and descriptions of subthemes

Theme and subtheme		Subtheme description
**Emotional challenges and stressors**		
	Lack of support for mental health	Difficulty in obtaining mental health support or resources for caregivers themselves or a lack of mental health resources for the caregivers themselves
	Physical health problems of family caregivers	The actual well-being and health of the caregiver as disabilities and other physical problems could hinder providing care for care recipients
	Balancing multiple responsibilities	Various and multiple responsibilities in personal and professional lives that do not directly encompass caregiving
	Evolving relationship with care recipients	Changes in dynamics between caregiver and care recipient because of dementia that can lead to conflicts or difficulties
**Functional challenges of daily living**		
	Lack of information	Difficulty in obtaining educational information related to caregiving for people living with dementia or specific to the care recipient’s stage of dementia to make decisions about older adult living arrangements
	Availability of resources	Difficulty in obtaining older adult living resources (eg, professional caregivers, facilities, and housekeeping) because of the scarcity of resources was mentioned
	Accessibility to resources	Difficulty in obtaining access to or receiving qualification for older adult living services or resources for the care recipient
	Affordability of resources	Situations where cost of older adult living resources or services for the care recipient was prohibitive or the costs were too high to be affordable
	Multiple caregiver dynamics	Situations or relationships that have influenced decisions made regarding caregiving for the care recipient because of caregiving responsibilities shared by multiple caregivers
**Challenges with paying for dementia care**		
	Hard to understand the financial procedures	Challenges associated with understanding the necessary financial aspects or procedures associated with caring for a person living with dementia
	Hard to navigate financial benefits programs	Challenges associated with navigating or using the materials or pecuniary support, programs, insurance, or other benefits that cover some costs of caregiving for people living with dementia
	High cost of caregiving	Challenges associated with financially supporting or paying for necessary caregiving resources or services for people living with dementia
	Difficulty in finding reliable financial professionals	Challenges associated with finding reliable financial professionals to seek advice or guidance from experts in financial aspects of caregiving for people living with dementia
**Legal challenges**		
	Setting up advanced directives	Written statement of care recipient’s wishes, such as will and powers of attorney
	Lack of information for legal procedures	Challenges associated with understanding the legal aspects or necessary procedures associated with caring for a person living with dementia, including preparing documents
	Difficulties in finding eldercare law attorneys	Difficulties encountered while attempting to find lawyers who specialize in eldercare law
**Expectations for a web-based platform**		
	Mental health support groups	Groups of caregivers of people living with dementia in similar circumstances who can provide emotional support and practical advice to each other
	Educational resources	Educational resources to educate caregivers on topics and useful practices related to dementia and caregiving for people living with dementia
	Information database to obtain services	A comprehensive database tailored to the needs of caregivers and care recipients for specific caregiving services, including older adult living services, financial services, legal services, and mental health
	Other qualities of a web-based platform	Any other qualities (eg, easy to use, esthetic, reliable, and interactive) or features (eg, communication tools and task-based reminders) of a web-based platform that should be included

#### Theme 1: Emotional Challenges and Stressors

All participants reported having some challenges when it came to caring for people living with dementia. These challenges included a lack of reliable guides and information on caregiving, a lack of support for caregivers’ emotional health, caregivers’ physical health issues, balancing multiple responsibilities, and evolving relationships and conflicts between caregivers and care recipients (Textbox 1).

**Emotional challenges and stressors.**

- Lack of support for caregivers' mental health
- Physical health issues with family caregivers
- Balancing multiple responsibilities
- Evolving relationships and conflicts between caregivers and care recipients

Many participants described caring for their loved ones as “stressful” and “overwhelming.” One participant noted a sense of guilt and emotional conflict:

You feel like a failure for having to put him somewhere...It is not easy to navigate all that. And you do have those yucky feelings in the back of your mind that you do not want to do this, but yet, you know you should...

PID030

Another caregiver reported the mental toll of caregiving:

Because she was no longer handling things the way she had previously been able to, I found it very challenging, trying to maintain a kind of calmness and peace and stability in our home.

PID0129

Caregivers needed to balance many other professional and personal responsibilities, such as working and caring for other family members, and this often led to significant stress. One participant said the following:

I do not have the mental capacity right now. I am trying to get tenure, and I have a kid, and I am trying...to sell my mom’s house...

PID003

Another participant mirrored the difficulties of balancing many responsibilities:

You know, trying to take care of my mom and then try to work a full-time job and then the other myriad other responsibilities. Things are pretty busy.

PID006

Another participant said the following:

Even though I work from home, to be able to take time off to attend a Zoom meeting or to go in person has not been feasible.

PID0129

Furthermore, caregivers were emotionally taxed by the evolution of their relationship with the people living with dementia as their loved one’s cognition and memory declined. These evolving relationships sometimes led to conflicts that were excessively stressful to resolve. With spouses, several participants expressed emotional distress, with one saying the following:

One day, he was going to bed. I said, “I will be up soon.” He turned around to me all seriously [and] said, “What does your husband think about this?” I was devastated. I thought, “Oh my goodness, he does not realize [he is my husband].”

PID058

Another familiar caregiver relationship that was often strained as the care recipient became increasingly dependent on the caregiver was the parent-child relationship. One participant said the following:

It is just hard seeing a parent on the slow decline...It is hard, as you can see, the cognition...It is sometimes sad. You know the person that you once knew is not the same person, but that does not make you love any less, but it tugs at your heart a little bit.

PID092

Another participant shared the strain on the parent-child relationship:

I see my mom and her behaviors that are all related to dementia. Sometimes, I have to have these walls that tell me it is not [that] she is not doing this to me, it is just the illness, so I do not take it personally.

PID018

#### Theme 2: Functional Challenges of Daily Living

Family caregivers often arranged living and functional assistance for care recipients. Some options usually included in-home professional caregivers; independent living arrangements; and senior communities such as assisted living facilities, nursing homes, or memory care. Furthermore, higher-acuity circumstances called for escalated care, such as that delivered in skilled nursing facilities and by palliative and hospice services. In a postacute illness situation (acute hospitalization, surgery, or significant illness), families may have needed to consider acute and subacute rehabilitation facilities, home health care services, and physical and occupational therapy agencies. Challenges that participants reported in finding suitable acute, subacute, and long-term living conditions included a lack of information on the types of assistance, difficulty comparing communities or support services, inability to pay or afford care, and difficulty assessing the quality of services (Textbox 2). For many participants, it was difficult to obtain educational information about the services offered by each type of entity and which types of services were recommended in different situations. This ambiguity affected the caregivers’ decision-making and led to stress.

**Functional challenges of daily living.**

- Lack of information on types of assistance
- Difficulty comparing communities or support services
- Difficulty assessing the quality of services
- Difficult obtaining information about types of services appropriate for different situations
- Inability to pay or afford care

One participant directly expressed these difficulties:

[It’s] been our experience when you look up assisted living facilities near me or whatever...If you check on one, there’s no real way to contact that one. I don’t even know where to begin. Do I even want to look at this facility if we can’t afford it?...You can’t get that information.

PID030

Another caregiver shared the difficulties of obtaining adequate information on older adult living services:

I was starting from nowhere and having to research...As for dementia, there’s not a lot of information because everybody’s different. Everybody, everyone, and every person with dementia have different meanings. So, it’s a little learning as you go.

PID113

When another participant was asked about the challenges of finding older adult living services, the caregiver responded as follows:

[I] feel lost. We feel like we don’t know what to do sometimes.

PID022

Another participant shared the difficulty of hiring reliable professional caregivers for the home:

Finding good caregivers was very difficult and a lot of time [was] having to manage the caregivers. [The caregiver] was as much work as having to help my mom and be there for my mom. There are many issues with the quality of care and people not showing up on time or things like that.

PID003

#### Theme 3: Challenges With Paying for Dementia Care

Family caregivers were also responsible for managing their older loved ones’ financial arrangements, often being the sole managers of the care recipient’s finances. In many cases, participants mentioned that the overall expenses for care, especially professional assistance, were too high, preventing them from initiating or maintaining such services (Textbox 3).

**Challenges with paying for dementia care.**

- High expenses for care and professional assistance
- Challenges in finding affordable care
- Difficulty obtaining financial assistance
- Difficulty utilizing benefits
- Confusion of management of property, estate, and funds

One participant shared that finding affordable care was the primary challenge regarding eldercare:

She needs someone with her pretty much most of the time. It’s something that we’re not sure we can afford yet. That’s the biggest thing is not being able to afford it.

PID022

Another participant shared the high cost of constant care for an older parent:

We have 24/7, 365 care for him. It’s costly, but he can’t be left alone for any real period of time.

PID021

Some caregivers immediately recognized their inability to pay for professional caregiving services, with one participant stating as follows:

I know that I financially could not pay for the cost of nursing home care.

PID058

Another financial challenge was caregivers’ or care recipients’ inability to qualify for certain financial health care benefits. One participant shared their difficulty using benefits:

He is a veteran. I’ve contacted the veteran association (VA), but you have to have a 70% or higher disability to gain access to their homes...He could eventually go on a waiting list, but the waiting list is long.

PID058

Another participant shared the difficulties of obtaining financial assistance because of specific qualifying parameters:

Medicare doesn’t step in and won’t help even with the in-home health unless she has a broken hip. And then they’ll help with that. But for dementia alone, they don’t want you to put them in a nursing home, but they also won’t let you keep them [at] home or [give] help.

PID064

Some family caregivers found the management of property, estate, and funds stressful and confusing. For many, understanding the process of setting up financial documents was a daunting task, as one participant said:

For me, the most challenging has been finding out what I needed to do, getting the right paperwork done.

PID109

One participant expressed the difficulties of navigating the financial aspects of caregiving:

That was hard. There’s just a lot of paperwork with being a power of attorney, the financial power of attorney, because every bank, every medical office, everything wants a copy of all this stuff...It’s just very daunting and time-consuming for a person that doesn’t know that going into it.

PID003

For many family caregivers, navigating insurance or other benefits that cover some caregiving costs was also challenging. One participant mentioned insurance policies:

Understanding the insurance and health things is overwhelming for me. I don’t like sitting down trying to read insurance policies...Trying to navigate through that is overwhelming.

PID093

Many participants expressed difficulties completing the necessary steps to qualify for financial assistance programs. One participant shared their experience with such difficulties:

I guess the biggest thing is I’m having some issues with her [Social] security. My father passed away in March. And when he passed away, she was supposed to get his social security automatically and they haven’t done that yet. And I’m still in the process of working on that...But with all the COVID and nobody having offices open, it’s been tough.

PID034

Family caregivers were usually responsible for providing additional financial support, including supplementing the care recipients’ resources (eg, savings, benefits, and insurance) when they were inadequate to cover the costs of needed services. One participant expressed the financial burden of caregiving:

[We pay] all out of pocket. There’s no help that I know, and I have to pay for this kind of stuff...You know, you’re always trying to calculate...What are we going to do if he lives beyond his money? It’s not a great position to be here.

PID021

Another participant discussed the high out-of-pocket costs:

We don’t have any long-term care insurance, and the costs are pretty high. It’s going to go into our savings.

PID027

#### Theme 4: Legal Challenges

Families and caregivers often must prepare legal documents such as advanced directives so that their loved one’s wishes can be communicated legally. Most participants admitted that setting up these advanced directives was a difficult decision and process that had to be made with family members and legal professionals (Textbox 4). One participant expressed the difficulties in setting up advanced directives:

I had spoken to my mother about, “Mom, you need to get these things in place.” And we need to take care of these things. And she would just put it off, so it would have been much easier for me if all of this had already been put in place.

PID129

**Legal challenges.**

- Challenges setting up legal documents including:
  Advanced directives & living wills
  Medical power of attorney
  Financial power of attorney
  Will / Probate 
  Guardianship 
- Difficulty finding lawyers specializing in eldercare law

Another participant shared an aspect of setting up advanced directives that was particularly challenging:

The most challenging [aspect] was all the forms need[ed] a notary, so you [got to] get everything and get everybody together to go someplace and have it notarized.

PID050

Owing to the process of obtaining the necessary documents, many participants found it time-consuming, with one participant stating as follows:

[It is] time-consuming...It just takes forever to get it done.

PID006

In addition, participants reported a lack of available educational information about estate and asset management, making it hard for family caregivers to understand the legal aspects or necessary procedures for securing a loved one’s belongings. Some participants shared the need for understanding such procedures:

Having the whole process more transparent would be phenomenal...It is convoluted and hard to understand, hard to figure out...

PID102

When addressing the challenges associated with asset management, one participant said the following:

It would be great to have that [professional legal advice], but I do not know how to find the help that I need in that area.

PID106

Other participants also reported difficulty finding lawyers specializing in eldercare law when they realized that they lacked an understanding of the nuances of eldercare law, thus needing specialized legal assistance. One participant found deciding between different recommended lawyers difficult:

Just trying to decide among the different ones that were recommended was probably the most challenging part.

PID027

Another caregiver noted the following:

...the choices for a lawyer are very slim. There are very few lawyers that do senior law. And you want one that does senior law. We found one who eventually helped us with getting the durable power of attorney, which is the financial aspect and the medical power of attorney.

PID064

In addition, those who hired eldercare lawyers found working with them stressful and shared their experiences:

Knowing when to call them, wondering, “Is this phone call going to cost? Are there add-ons that my current attorney has told me about?” And she put everything in the hands of her paralegal, which I am only [able] to communicate with through email, so I try synthesizing my questions and thoughts and only write one email at a time.

PID110

#### Theme 5: Expectations for a Web-Based Platform

As the participants discussed the challenges mentioned in previous sections, they expressed their expectations of a future digital solution and how it would ideally assist caregivers of people living with dementia (Textbox 5). There was evidence of a need for a comprehensive digital platform that integrates mental health support, educational resources, an information database on older adult care services, and other quality features. One participant expressed the need for such a comprehensive digital platform:

To have one place that would say, “Have you thought about this? Have you thought about this?” would be helpful.

PID030

**Expectations for a web-based platform.**

- A comprehensive database of commonly needed professional services
- Mental health support and caregiver support groups
- Educational resources on dementia and caregiving
- A platform that is easy to use, aesthetic, reliable, and interactive

Many participants reported that mental health– and caregiver-specific support groups on the digital platform would help them and reduce the dearth of mental health care available for caregivers of people living with dementia. They mentioned that caregiver groups could provide emotional support and practical advice to each other when experiencing similar situations. One participant expressed the need for online support groups:

I feel like a support group where you can communicate as much as you want...Then have a place to ask question[s]. That would be beneficial.

PID003

Another participant shared the need for self-care support:

Finding support groups is critical. We have to take care of ourselves before we take care of others.

PID106

Another participant shared the need for localization of these web-based support groups saying there should be *“*a chat function so you can connect with others from your area...the ability to connect with local people” (PID064).

Participants also would like a digital platform to provide caregivers with educational resources on dementia and caregiving for people living with dementia. Caregivers requested an increased number of web-based educational topics related to dementia that would be helpful in providing information on and good practices related to dementia and caregiving for people living with dementia. Some participants believed that these educational resources would be helpful in guiding them on the decision-making process, especially because there was a lack of information and understanding regarding dementia progression, older adult care, securing older adult living arrangements, and understanding the financial and legal processes involved. One participant expressed the importance of educational resources:

I think educating people on what is coming next is something that would at least help me. So, I know she is going to lose her memory, and she is not going to be able to remember anything or 90% of things. It is like mentally, physically, and around the house, I can now prepare for that. I have got to be able to think [about], this is what I am doing for them right now, [and] this is what it is going to be [in the future].

PID106

Other participants also shared that an educational resource on the progression of dementia would be helpful, such as one participant who stated as follows:

A timeline of progression in terms of someone’s dementia...so I can have a view of the future.

PID050

One participant summarized their ideal digital platform for caregiving by saying the following:

We need a handbook on this saying, “Okay, you have got someone you love who has been diagnosed with dementia.” Or even start before that, where “I think my loved one might have dementia” and have a checklist of things to do next...I want a manual [on] how to do this [caregiving].

PID102

There was widespread consensus among participants that a comprehensive database of commonly needed professional services would help caregivers and care recipients find older adult living communities, home care agencies, financial services, legal assistance, and mental health services. One participant said that a valuable aspect of a web-based platform would be “a database of available services. I think it would be great if there were some ways just to say, you know how I can get help with this, like getting [transportation for] my mom to the doctor” (PID007). Other participants expressed the desire for a system to review and compare caregiving services and said that there should be a functionality that allows caregivers to leave “*...*feedback or comments or reviews [from] people who work in the industry and their families who used the service” (PID003).

Participants also expressed their expectations regarding other qualities of a web-based platform. Many participants hoped that the web-based platform would be easy to use, esthetic, reliable, and interactive. One participant said that the platform should be “easily navigated by those of us who are not that tech savvy” (PID030). As they had limited time, several participants said that the web-based platform should be easy to use and understand. One participant said the following:

Just some of that stuff [in] more in plain language, where people do not have to research so much because it takes so much time [to research].

PID113

## Discussion

### Principal Findings

This study focused on the legal, financial, and functional challenges of caregiving for a person living with dementia. Our thematic analysis of interviews with caregivers of people living with dementia showed that they are an underresourced group with widespread struggles as they balance a multitude of responsibilities, including familial obligations, work, financial burdens related to care, finding reliable services and assistance, navigating legal necessities, and seeking emotional support. To address these challenges, caregivers shared their expectations for a future digital tool that could help relieve some of their caregiving burden. Family caregivers reported their expectations for a comprehensive and easy-to-use digital health platform where they could search for educational and caregiving information; seek support from peer caregivers; and find reliable medical, financial, and legal professionals. Our results suggest that a comprehensive database is needed to locate reliable living facilities, find financial and legal professionals to set up documents, obtain timely advice from health care professionals and dementia experts, manage caregiving-related stress through support groups, and prepare for specific caregiving tasks by providing organized reminders and relevant caregiving education.

Following previous findings [21-23], our study confirmed that family caregivers face a variety of challenges, including a lack of mental health support, difficulties balancing multiple responsibilities, and a lack of information about various services. In addition, consistent with previous studies [8,24-26], caregivers reported limited availability, accessibility, and affordability of resources and difficulty finding reliable legal and financial professionals. Furthermore, we found that caregivers expressed concern about the insufficiency of current social benefits programs to cover the costs of caring for a loved one, underscoring a significant public policy landscape and indicating the need to develop, implement, maintain, and evaluate additional caregiver support programs [27].

In addition to supporting previous literature, there are several ways in which this study contributes to a current research gap. First, we found that the relationship between caregivers and their care recipients and the dynamics among multiple caregivers are significant factors in the care decisions of people living with dementia. Therefore, eldercare services must consider engaging families in a holistic decision-making process that accounts for multiple family caregivers and diverse stakeholders [28,29]. Furthermore, it is difficult for family caregivers to locate readily available and reliable information on the web about older adult living options and functional care, legal procedures, and navigating financial benefits and professionals. This indicates the unmet needs of family caregivers and the significant demand for comprehensive interventions and programs that provide information and assistance in navigating these processes and services. Therefore, solutions must be developed to support caregiving for people living with dementia and resolve conflicts between caregiving and work.

Owing to the various demands on their time, many caregivers face conflicts between family and work obligations that, in turn, may lead to possible work-related strain and a decrease in caregiving performance and quality [30]. Providing care for people living with dementia has also been reported to negatively affect caregivers’ physical, mental, and social self-care [31], indicating the need to provide educational resources about self-care practices and a platform to connect caregivers faced with similar challenges. Therefore, providing care support such as task-based reminders, mental health support, and aid in locating professional services would be particularly beneficial to relieve the stress associated with balancing work and caregiving. This study also revealed that many family caregivers hoped that the digital tool would be easy to use, esthetic, reliable, and interactive. These findings may provide insights into the future development of digital platforms seeking to engage family caregivers of people living with dementia as target users.

It is critical to develop a digital platform that provides helpful and usable information to caregivers of people living with dementia. To provide more sophisticated care recommendations and support to caregivers of people living with dementia, AI and large language models can be used to offer a tailored yet comprehensive experience to these users. Large language models are highly efficient at processing large amounts of data, which can be used to sort through thousands of care options informed by the needs, location, and financial situation of the care recipient to find the right care solutions. To ensure that the AI-powered personalized care-matching model offers accurate and safe recommendations, quality control and standardization of the data used to train the model are critical. In addition, expert supervision should be used to validate and improve the accuracy and quality of the results. Finally, such a model must be regularly trained and updated using new data to ensure that it is current and accurate.

### Limitations and Strengths

This study has several limitations. It was conducted via Zoom with relatively few participants, who tended to be well educated and had access to technology. As a result, this study may not reflect the full range of caregiving experiences. Relatedly, the participants’ characteristics may not be representative of all family caregivers of people living with dementia. In addition, there may be biases in the analysis and interpretation of the study findings as 2 primary coders had varying degrees of direct experience providing care to people living with dementia. Finally, because of the focus of the analytical framework and predetermined research questions, as well as the potential limitation of using saturation as end point for recruitment [32], other personal caregiving experiences and participants’ perspectives may be yet to be shared in full detail.

However, there are unique strengths to this study. First, this study reached data saturation based on group discussion and analysis [18], suggesting that the study findings summarized the perspectives of the study sample comprehensively. Second, participants were diverse regarding their socioeconomic characteristics, such as age and occupation, so their perspectives were valuable and applicable to many people. Most participants in this study were female (23/30, 77%), non-Hispanic White (27/30, 90%), and aged >50 years (25/30, 83%). This demographic composition aligns with the findings of the 2020 Caregiving in the U.S. report [33], which also highlighted that >50% of family caregivers share such demographics. Furthermore, the study team was composed of interdisciplinary researchers, multigroup coding was conducted, discussions regarding the analysis and results were thorough, and participants were fully engaged in the design and reporting of the study. Therefore, this study is one of the few studies carried out using a participatory-based approach [17] and has incorporated the theory of community-based research [34].

### Conclusions

This study re-emphasizes the legal, financial, and functional challenges that caregivers face in providing care to persons living with dementia. All participants acknowledged challenges in their caregiving roles. Realities such as caregivers’ physical and financial limitations were shown to exist within the context of many other challenges, demonstrating a complex, intersectional environment for family caregivers. Specifically, the most common challenges included balancing other family obligations and work, managing financial burdens, finding reliable services, navigating the legal or financial process, and seeking emotional health support. This offered a comprehensive view of this population’s current challenges and a clear vision of potential resources and interventions that may support caregivers. Notably, participants expressed a need for a comprehensive digital platform. It was identified that such a platform should integrate mental health support, educational resources, an information database on older adult care services, and other quality features. Such a platform, coupled with a further comprehensive analysis of the struggles and limitations of current caregiver support, has the potential to assist this population of caregivers of people living with dementia significantly in their role.

## Appendix

Multimedia Appendix 1 Adapted caregiver interview script guide.

## Abbreviations

AD: Alzheimer disease
ADRD: Alzheimer disease–related dementia
AI: artificial intelligence
IRB: institutional review board
