# Supplementary material for: Financial, Legal, and Functional Challenges of Providing Care for People Living With Dementia and Needs for a Digital Platform: Interview Study Among Family Caregivers
Source: JMIR Aging. 2023 Sep 5;6:e47577. doi: 10.2196/47577 (PMC10509746; doi:10.2196/47577)
Supplement: Multimedia Appendix 1 [file aging_v6i1e47577_app1.pdf]

Caregiver Interview

Introduction

- Hello, firstly thanks for participating in the CARES study
- The goal of this interview is the understand the needs and key areas of concerns for family caregivers of people living with dementia
- Findings from our conversation will be used to inform the development of a personalized support served that connects family caregivers to eldercare professionals and educational resources
- We expect this to take roughly an hour
- Overview of interview

Verbal recap of the study

- We will conduct a **series of three interviews**, this will be first over the next 5 months or so - we'll follow up with more details
  - The first interview will be to understand the needs of family caregivers
  - Then use this feedback to develop a solution, and in our second conversation will be a discussion of how well a preliminary version of that solution functions. That will be the second interview
  - Final the last interview will be an final evaluation of the developed solution
  - So we want to learn, use that information to build, and then test

Informed Consent

Do you mind us recording

- **General**
  - This was the form that you agreed to at end of the survey; it's a four page document so we'll focus on the key aspects, which should take too long
- **Key information**
  - Your participation is completely voluntary - you can choose to not be part of this study at any time
- **Why are you being invited to take part in the study?**
  - Fristly you are a resident of Texas
  - You providing care for a family member living with dementia
  - You are not being paid are a professional caregiver
  - And you have concerns about or need for more information on financial legal aspects of caregiving.

- **[Don't say] What happens to the information collected for the study?**
  - But your identifiable information will be stored separately from other study data and linked using a unique identification code
  - This identifiable information will only be accessible to the authorized study staffs

created with craft

- While no identifiable inforation will be shared, the study data, could be used for future research studies or distributed to without additional informed consent
- The study data, without identifiable information, will be stored electronically in access-limited folders at Olera Inc. and Texas A&M University for at least 3 years from the end of the study.

[Were almost there I promse]

- **[Don't say] Do I get anything for participating?**
  - You will be receiving a $25 stipend for your participation in the testing, interviews, and surveys, which will be provided via check by mail
  - But remember that there is a series of three interviews
  - If we don't recieve a response from you for three weeks or if you fail to participate in at least 3 interviews, then you will be deemed not complying and none or partial stipends may be provided.
- **Risks associated with this study involve**
  - Time and inconveniences from participating in the consent discussion and the rest of the study
  - A possible risk is also privacy breach, but our team has a well developed procedure to keep your information safe and your name is not tied to the information that we record, so likelihood of this is low
  - Besides the giftcard, there are not benefits of the study. Although participation in this study may include:
    - Increase in your awareness of relevant resources;
    - Increase in your understanding of caregiving concepts
    - Of course how to navigate online caregiver support platforms like ours

- ## Before we proceed to the interview, do you have any questions?

Interview Questions

Introductory questions

1. **Comment:** Just to get started, we like to know about your experience being a caregiver for someone living with dementia. **Question:** In general do you mind sharing your experience?
2. What kinds of services do you think would help you?
3. [Also] Please let us know how you would like us to refer to this person? (e.g name)
3. Comment: Let's talk about elder care services in detail. We are particularly interested in your experience with three categories of services and professionals:
   1. Elder living (which include nursing homes, assisted living facilities, in-home care, etc.)
   2. Financial services (examples: insurance experts, financial managers/planners, care managers, etc.)
   3. Legal services or professionals (examples: attorneys, legal experts, etc.). Question: What were/are the most critical services, professionals or businesses/agencies that help/helped you with caregiving responsibilities?

Elder living questions

created with craft

1. To begin with elder living services
1. What was the most challenging aspect of finding and securing living arrangements for your care recipient?
2. How did you identify and compare elder living facilities or in-home care agencies? (e.g., online, doctor referral, friend/family referral)
3. What information is/was most important to you when choosing a service provider, facility, or business/ agency to help you secure living arrangements for your care recipient? (Examples: price, location, services provided, type of accepted insurance, etc.)

Finances

1. Comment: Let's talk about another specific type of service. We are interested in more in-depth understanding about financial services that help families with caregiving responsibilities.
2. What was the most challenging aspect of navigating the finances aspects of caregiving?
3. Did/do you consult with any financial professionals to help you with caregiving responsibilities? If not, why not? If so, what kind of professionals and how did you select them?
4. Are you aware of the types of financial support, benefits, and resources available to you and your loved one?

Legal services questions

1. Did you consult with any legal professionals on eldercare legal matters? If not, why not? If so, how did you select these services?
2. Did you or any members of your care team set up advanced directives for your loved one? If so, can you describe the process?
3. What was the most challenging aspect of navigating the eldercare legal process during your caregiving?

Conclusion

1. What caregiving support groups or online forums do you participate in if any?
2. What education on dementia caregiving would you find beneficial? (e.g., are there any topics you wish you knew more about to better complete caregiving tasks?)
3. If you could have a "magic wand" to create an online tool/platform that would assist you with your caregiving, what kind of features will it have?

Next steps

1. Our team will take this discussion and translate it into a solution
2. And we want your feedback on what we develop
3. We will follow up with you on next steps

created with craft
